# Overturning *Roe v. Wade*: consequences for midlife women’s health and well-being

**DOI:** 10.1186/s40695-022-00085-8

**Published:** 2023-01-06

**Authors:** Judith A. Berg, Nancy Fugate Woods

**Affiliations:** 1 The University of Arizona College of Nursing, 800 The Mark Lane, San Diego, CA 92101 USA; 2 Biobehavioral Nursing and Health Informatics, University of Washington, 4525 E Laurel Dr NE, Seattle, WA 98105 USA

**Keywords:** Abortion, Contraception, Women, Midlife health

## Abstract

In July 2022, the United States Supreme Court issued a decision in *Dobbs v. Jackson Women’s Health Organization* that overturned *Roe v. Wade,* the Supreme Court decision that legalized access to abortion in the United States. The overturning of *Roe v. Wade* means women’s ability to choose to have an abortion or continue a pregnancy is no longer protected by the constitution of the Unites States (Rohan, Editorial: Overturning Roe v. Wade: What are the implications for perinatal nurses?, 2021). Consequently, each state can now independently decide the legality of abortion. The purpose of this commentary is to discuss potential consequences of the overturning of *Roe v. Wade* for women’s health, particularly midlife women’s health, in the United States. The consequences discussed include unintended pregnancy, access to the full array of reproductive health services including abortion, impact on the reproductive health of poor women and women of color, and the impact on midlife women in their caregiving roles.

## Background

In July 2022, the United States Supreme Court issued a decision in *Dobbs v. Jackson Women’s Health Organization* that overturned *Roe v. Wade,* the Supreme Court decision that legalized access to abortion in the United States. The overturning of *Roe v. Wade* means women’s ability to choose to have an abortion or continue a pregnancy is no longer protected by the constitution of the United States (US) [23]. Consequently, each state can now independently decide the legality of abortion, and according to the Guttmacher Institute, in 2022 26 US states had multiple bans ready to enact, 13 of which were trigger laws that automatically took effect when *Roe v. Wade* was revoked [13]. Eleven states had early gestational age bans, meaning that abortion was not allowed past the first trimester or another time point in the pregnancy [13]. At the same time, there are 15 states and DC that have laws in place that are protective of abortion. Of those 15, Maryland, Connecticut, and California introduced more extensive protections in anticipation that women from the 26 states with abortion bans would travel to the protective states for services [26]. The purpose of this commentary is to discuss potential consequences of the overturning of *Roe v. Wade* for women’s health, particularly midlife women’s health. The consequences discussed include unintended pregnancy, access to the full array of reproductive health services including abortion, impact on the reproductive health of poor women and women of color, and the impact on midlife women in their caregiving roles.

## Unintended pregnancy and access to full array of reproductive health services

The possibility for pregnancy remains until women achieve menopause [16]. Midlife women may be confronted with an unintended pregnancy, which is defined as a pregnancy that occurs when a woman does not wish to be pregnant. For this manuscript, midlife, the period of the lifespan between younger and older adulthood, is defined as women 40 to 65 years of age [32]. A 1995 survey revealed 51% of pregnancies among women 40 and older are unintended (Sherman, Harvey & Noel, [27]). Therefore, the negative consequences of the reversal of *Roe v. Wade* are likely to be highly relevant to women in midlife. In a systematic review of unintended pregnancies in US women, Aztlan-James and colleagues found factors associated with multiple unintended pregnancies to be increasing age, identity as Black or Hispanic, having a below poverty level income, experiencing a non-voluntary first sexual intercourse especially at a young age, participating in the sex trade, experiencing stressful life events, and having had a previous abortion [4]. It should be noted sexual health of midlife women often is overlooked by health care providers despite increased rates of sexually transmitted infections and unintended pregnancy during this part of the lifespan [30]. Many women in their 40 s become pregnant because they incorrectly believe reduced fertility means they no longer need contraception [29]. With little attention to midlife women’s sexual health, contraceptive needs may go unaddressed and unintended pregnancy will be more likely to occur. Alternatively, midlife women might be told they are no longer fertile when, in fact, fertility can be erratic for years before women stop ovulating completely. Some authorities recommend women under 55 continue to use contraception if they do not want any more children [29].

New data from the Guttmacher Institute depicted an increase in abortion numbers from 2017–2020. This demonstrates a reversal of the long-term decline in abortions in the US that began 30 years ago [19] and was consistent in all four regions of the country. The implication is that overturning *Roe v. Wade* comes at a time when need for abortion care is increasing. The impact of the loss of *Roe v. Wade* may be more damaging than predicted especially in areas of the country where access to abortion care is already a struggle. For example, the 930,000 abortions obtained across the US in 2020 represents a sustained increase in abortion and more than one in three of those abortions were obtained in states that are now certain or likely to ban abortion [14]. As well, decades of research consistently show that abortion bans and restrictions don’t reduce unintended pregnancy or the demand for abortion, and do not improve women’s health. Instead, they impose significant hurdles to obtaining care, causing stress for people in need of abortion and leading some to experience forced pregnancy with all its consequences [15]. The Guttmacher data were not disaggregated by age, but abortion surveillance data [20] reported in November 2022, for 2020 suggested a 2% increase in the total abortion ratio but decreased abortion ratio in women aged ≥ 40 years compared with other age groups. Yet, a total of 22,407 abortions were reported in the US for women aged ≥ 40 years in 2020, representing a significant number of midlife women affected by unintended pregnancy who opted for pregnancy termination.

With the loss of *Roe v. Wade*, women of reproductive potential (menarche to menopause) in states that restrict or completely ban abortion likely will face critical access issues. Abortions probably won’t stop, but global data indicate that they could become less safe [28]. Global information gleaned from scientific literature, non-governmental organizations’ websites and online discussions combined with statistical modelling estimated how many abortions are safe (done with a method recommended by the WHO), less safe (done by a trained professional using an outdated method or without sufficient information or training) or unsafe (done by an untrained person using a dangerous method). These researchers estimated that almost 90% of abortions in countries that allow abortion on request are safe, but abortions that are categorized as less safe and least safe are much more prevalent in places where abortion is restricted [28].

Overturning *Roe v. Wade* has significant implications for medical and nursing education and will reshape the knowledge, skills, and quality of care provided by future physicians and nurses [33], particularly in states with bans on abortion or pregnancy age restrictions. Medical school graduates without abortion training will be limited in the skills necessary to manage pregnancy complications including placental abruption, infection, ectopic pregnancy, and eclampsia, because the same medications and surgical techniques utilized for abortion also treat obstetric complications [33]. Moreover, without adequate abortion care education, long-term quality of reproductive healthcare in the U.S. will likely deteriorate, with negative consequences for women’s health [33]. At the same time, maternal-child nurses (and other health professionals) have long supported policies to reduce maternal mortality, promote health equity, eradicate structural racism, protect the patient-provider relationship, and improve health care delivery for all. With the loss of *Roe v. Wade* many of these basic policies will be undermined. Therefore, nurses must be educated and trained to care for an increased number of women with delays in seeking prenatal care, as well as with pregnancies complicated by unsafe abortion attempts, that might include drinking toxic fluids; ingesting teratogenic or labor-inducing herbs; inflicting direct injury to the vagina, cervix or rectum; or repeated striking of the abdomen. According to the World Health Organization, over 2.5 million unsafe abortions occur each year and are responsible for pregnancy complications associated with nearly 8% of maternal deaths (Say et al., [24]). Even with heightened surveillance by nurses, the question becomes whether or not physicians, physician assistants, and advanced practice nurses will be adequately educated and trained to manage serious abortion complications.

Health risks associated with pregnancy in midlife women may be life threatening, particularly because health care provider education may not include abortion care and management of complications. For example, higher maternal age has been associated with spontaneous abortion, fetal death or stillbirth, and ectopic pregnancy [2]. The risk of spontaneous abortion was 8.9% in women aged 20–24 years and 74.7% in women aged 45 years or more. The overall risk of ectopic pregnancy was 2.3% but showed a steady increase in incidence with increasing maternal age at conception from 1.4% of all pregnancies at the age of 21 years to 6.9% of pregnancies in women aged 44 years. The overall risk of stillbirth was 4.3 per thousand women, and though an increase in stillbirth was associated with maternal age, the association was less than for spontaneous abortions and ectopic pregnancies [2]. In a more recent study, the risk of fetal loss was higher among women 35 years and older compared to younger women. Influencing factors for fetal loss in women with advanced maternal age (over age 35) were low educational level, unemployment, abnormal pregnancy/labor history, and pregnancy complications [34]. The consequences of miscarriage later in pregnancy, in the absence of access to abortion, may prevent women from obtaining necessary care to insure their best mental and physical health. As well, lack of access to abortion may force a woman to carry a dead fetus longer than necessary, because the procedure for treatment for miscarriage and abortion are the same [6].

As women age, they are more likely to have needs that require ongoing medical care, especially because chronic health conditions increase risk for pregnancy complications. These include autoimmune diseases (ankylosing spondylitis, inflammatory bowel disease, multiple sclerosis, psoriasis, rheumatoid arthritis, scleroderma), conditions that affect the blood, blood vessels, heart and lungs (asthma, heart disease, high blood pressure, human immunodeficiency virus [HIV]), conditions that affect hormones (diabetes, thyroid conditions), and mental health conditions that interfere with daily life, such as depression [21]. Similarly, women with a history of cancer or any chronic condition may be at serious increased risk during pregnancy. In states with abortion bans, these patients may be told they cannot obtain an abortion until their life is in jeopardy [36]. A health care workforce untrained in managing pregnancy and/or abortion complications may contribute to the health risks of midlife women, especially if these women are by circumstances, unable to terminate an unintended pregnancy.

Some women with unintended pregnancy living in states with abortion bans will be able to travel to states that are protective of abortion, but that will be limited to women who can afford it. Midlife women are likely to already have children, and women with children will have the additional problem of how to care for those children left behind during the travel period. Although not yet in place, some have rumored abortion foes hope to prosecute women who travel to obtain abortion and even reward “tattle tales” who report women who have travelled to obtain abortion care. Meanwhile, in August, 2022, CBS News announced California is launching a new fund to help women from other states travel there for reproductive care. The state is prepared to spend $20 million to bring women from other states to its abortion clinics [5]. While this fund is intended to increase access for women residing in states with abortion bans or restrictions, single mothers and mothers unable to find childcare for existing children will likely not be able to take advantage of the available money. Furthermore, there is uncertainty about the capacity of providers and clinics in abortion protective states for providing abortion care to additional numbers of women from states that ban abortion.

Having a safe abortion is not necessarily dangerous or harmful to women, but being denied an abortion may be [18]. Researchers in 2016 reported that compared with women who were able to have an abortion, those forced to complete their pregnancies were more likely to suffer depression or anxiety disorders five years later [9] and had poorer physical health [22]. As well, a 2018 study found that after a woman who already had young children sought but was denied an abortion, the existing children were slightly more likely to have lower developmental scores and to live below the Federal Poverty Level than the children of women who received a wanted abortion [12]. In another study, two of the 161 women who were denied an abortion died of causes linked to pregnancy or childbirth. None of the women who received abortions died of pregnancy or childbirth-related causes over the following five years [22]. This is a 100 fold higher rate of mortality than expected (2/161 = 1243/100,000 MMR). Rates of maternal death have been rising in the U.S., and it’s now close to one death per 1000 cases of childbirth. That rate of 0.1% is the highest rate in the industrialized world, and even higher among women in the study who were denied a wanted abortion (1.2%) [18]. These data do not support policies that restrict women’s access to abortion on the basis that abortion harms women’s mental health [9] and/or physical health [22].

## Health of poor women and women of color

Evidence shows the disproportionate and unequal impact abortion restrictions have on people who are already marginalized and oppressed, including Black and Brown communities, other people of color, people with low incomes, young people, LGBTQ communities, immigrants and people with disabilities [15]. This discussion focuses on poor women and women of color. Forty-nine percent of abortion patients have an income below the poverty line, according to the Guttmacher Institute [25]. In 2019, almost four in ten abortions were among Black women (38%), one-third were among White women (33%), and one in five among Hispanic women (21%), and 7% among women of other racial and ethnic groups [3]. Therefore, more than half of abortions were among women of color prior to the overturning of *Roe v. Wade*. Potential reasons why abortion rates were higher among some women of color include their more limited access to health care, which negatively affects access to contraception and other sexual health services that are essential to pregnancy planning. In addition, the US health care system has a history of racist practices targeting the sexual and reproductive health of people of color, including forced sterilization, medical experimentation, the systematic reduction of lay midwifery, and discrimination by individual providers including dismissive treatment, assumption of stereotypes, and inattention to conditions, such as fibroids, that take a disproportionate toll on women of color [3]. Social determinants of health (income, housing, safety and education) affect decisions related to family planning and reproductive health. Poor women and women of color between ages 18–49 face greater barriers to accessing health care overall compared to their White counterparts, particularly for those with Medicaid for health insurance which has limited coverage for abortions. The Hyde Amendment has prohibited the use of federal funds for coverage of abortion under Medicaid except in cases of rape, incest, or life endangerment for the pregnant person [3]: even before the loss of *Roe*, these women had more limited access to abortion care. Plus, federal restrictions have chipped away at comprehensive, evidence-based family planning supported by the Title X program that has historically supported women and families in need [31].

Many women of color have more limited financial resources and transportation options than White women, which would make it more difficult for them to travel out of state for an abortion. Plus, out of state travel is likely to raise the cost of abortion due to added costs for transportation, accommodations, and childcare. Vehicle access is more limited among women of color; Black women ages 18–49 are over three times as likely as White counterparts (13% vs 4%) to live in a household without access to a vehicle and Hispanic women are more likely than White women (6% vs 4%) to lack access to a vehicle. Coupled with the childcare needs of existing children, limited transportation options may truly make it impossible for these women to travel for services. There may also be more missed work, meaning loss of pay, increasing the economic cost of abortion. Again, the effect will be felt more profoundly by women of color and those with lower incomes [11]. Further, current employment may make it impossible for women to leave work for the length of time it takes to travel to a state that has abortion care access.

Overall, the loss of *Roe v. Wade* is predicted to limit poor women and women of color’s access to the full array of reproductive health care, including abortion services. Their restricted access is likely to be a function of no insurance or health insurance that does not cover reproductive health and abortion care, and the high cost of seeking abortion care out of state. Moreover, some states with abortion bans that predate *Roe v. Wade* had Trigger Laws that went into effect simultaneous to the overturning of *Roe*. The heavy concentration of states in the South that restrict or ban abortion have a greater impact on communities of color, because more than half of all US Black women and a high proportion of Latina women live in the South [17]. These groups face disproportionate hardship in attaining abortion care [11]. The outcome may be that poor women and women of color are forced to continue with an unintended pregnancy that may expose them to greater health risks known to accompany pregnancy.

## Impact on midlife women in their caregiving roles

Much has been written about society’s expectations of women as caregivers, particularly unpaid caregivers (informal) in midlife [7, 8]. Society assumes women will take on the caregiving for children, spouses, parents, other relatives, or a mix of those. Three out of five caregivers in the U.S. are women and on average, caregivers of adults are 49.4 years old [1]. Half of all women caregivers work outside the home, and some have an older adult needing care living in their home along with minor children [35]. Yet, little is known about caregiving stress and unintended pregnancy in the caregiver. Uncovering this issue is now incredibly important since the overturning of *Roe v. Wade*, as access to reproductive health care and abortion services is likely to become increasingly difficult for caregiving midlife women. We’ve already discussed the numbers of unintended pregnancies in midlife women and must also consider implications of unintended pregnancy in the context of the multiple roles that midlife women perform simultaneously. For example, a single midlife woman who is working full time to support her children and her household may also be responsible for an adult relative or relatives living inside her home. She may reside in a state that has imposed bans on abortion care. In the event she encounters an unintended pregnancy, her ability to travel to a state that is protective of abortion is seriously restricted due to employment issues, financial needs, and her caregiving responsibilities. The likelihood of her being able to absent herself from employment and caregiving responsibilities may be all but impossible. Therefore, this midlife woman may face continuing an unintended pregnancy simply because access is not possible. Should this woman also live in poverty and be of color, she may face serious health issues during her pregnancy.

In another scenario, a midlife woman may have a teenage daughter with an unintended pregnancy. Again, if she is living in a state that has abortion bans in place, the ability of her daughter to travel for abortion care has many contingencies: can the daughter travel alone, can the midlife mother accompany her daughter, can they afford the expenses of travel and abortion services, do they have access to a vehicle in which to travel, and can the midlife mother leave other children at home, be away from her employment, and leave her caregiving responsibilities unattended? Should it not be possible for the pregnant daughter to obtain abortion care, the midlife mother may find herself in a position to be the caregiver of a grandchild—on top of her already multiple responsibilities. All of these issues reflect women’s lived experiences, particularly for poor women and women of color; but they are also particularly relevant to all midlife women who have a daughter with an unintended pregnancy.

## Conclusion: how to alleviate consequences of overturning Roe v. Wade

As we begin a new year it is disheartening and infuriating to see the imposition of legal limitations in the United States to women’s agency of our own bodies. Absent social policies supporting access to health care and early childhood education and caregiving, disparities in women’s access to abortion creates disadvantageous conditions for both midlife women and future generations of children born as the product of an unwanted pregnancy. It is time to recognize the ethical implications of this decision for people with and children of unwanted pregnancy. Opportunities to reverse the consequences of the SCOTUS decision [10] to limit access to abortion should motivate responses among advocates for women’s and children’s health and well-being, including imagining strategies for an equitable society in which all children have rights to a healthy life.

Until policy changes that support abortion access are enacted overall in the U. S., strategies that can be implemented immediately include:

- Strengthen sexual health education provided in schools to increase awareness of options for preventing unwanted or unplanned pregnancy;
- Improve access to information about fertility and fertility management, including monitoring menstrual cycles and menopausal status, and reliable and effective birth control approaches for all reproductive life stages, with attention to the specific needs of midlife women;
- Advocate for inclusion of explicit coverage of effective and reliable contraception and other pregnancy prevention methods in health insurance plans;
- Provide readily accessible and affordable early pregnancy detection;
- Provide “morning after” contraception to women to have on hand “just in case” (Plan B);
- Advocate for policies that would insure women’s right to agency over our bodies.

It is essential that women’s health advocates actively engage in political debates and policy dialogues that are intended to improve access to the full array of sexual and reproductive health care for all women. Now is not the time to remain silent; it is time to once again advocate for policies known to improve health and support women’s self-determination.

## Data Availability

Not applicable.

## Abbreviations

US: United States
